# Eosinophil-associated matrix remodeling in a sterile granulomatous inflammation model: a temporal histopathological analysis

**DOI:** 10.1007/s00418-026-02505-6

**Published:** 2026-06-25

**Authors:** Bruno Marques Vieira, Milla Bezerra Paiva, Juliane Siqueira Francisco, Rebeca Sousa Brum, Lucas Everton Simões, Maria Ignez Capella Gaspar-Elsas, Pedro Xavier-Elsas

**Affiliations:** 1 Laboratório de Citocinas, Department. of Immunology, Instituto de Microbiologia Prof. Paulo de Góes, Universidade Federal do Rio de Janeiro, Rio de Janeiro, Brazil; 2 Laboratório de Biomedicina do Cérebro, Instituto Estadual do Cérebro Paulo Niemeyer (IECPN), Rio de Janeiro, 156, Centro, Rio de Janeiro, 20231-092 Brazil; 3 Laboratório de Medicina Experimental e Saúde, Instituto Oswaldo Cruz, FIOCRUZ, Rio de Janeiro, Brazil; 4 FIOCRUZ, Rio de Janeiro, Brazil

**Keywords:** Eosinophil, Granulomatous inflammation, Matrix remodeling, Fibroplasia, Foreign body reaction, Histopathology

## Abstract

Eosinophils are increasingly recognized as contributors to tissue remodeling and chronic inflammation, yet their spatiotemporal behavior in sterile granulomatous reactions is incompletely defined. We performed a day 1–14 histopathological time course of a reproducible sterile granuloma induced by implantation of a heat-coagulated egg white pellet into the murine peritoneum. The reaction showed a biphasic inflammatory pattern with early neutrophil predominance followed by a sustained eosinophil-rich phase that persisted through late remodeling. Across time points, lesions progressed from exudative inflammation with prominent fibrin to organized fibroplasia and neovascularization, culminating in collagen-dominated encapsulation by day 14. Eosinophils concentrated at the pellet–tissue interface, frequently displaying apoptosis and degranulation, and occurring in close spatial association with fibroblasts and macrophages. At late time points, the outer granuloma zone contained mononuclear phagocyte/macrophage-like cells alongside dense connective tissue maturation. Together, these findings provide a temporal histopathology atlas of sterile implant granuloma maturation and introduce a semiquantitative framework that summarizes key tissue-composition transitions (fibrinous exudate → fibroplasia/collagen encapsulation) alongside eosinophil enrichment at the pellet–tissue interface.

**Supplementary Information:**

The online version contains supplementary material available at 10.1007/s00418-026-02505-6.

## Introduction

Eosinophils are multifunctional leukocytes implicated in host defense and in a broad spectrum of inflammatory and fibroproliferative processes, and in physiological and pathological processes (Rothenberg and Hogan 2006; Lombardi et al. 2022). Their roles in tissue remodeling, immune modulation, and granuloma formation have attracted increasing attention, particularly in chronic inflammatory conditions. However, despite their recognized importance, the study of eosinophil biology remains challenging because of the limited availability of well-characterized experimental models that reliably induce and sustain eosinophilic inflammation (Jacobsen et al. 2012; Allen and Sutherland 2014). In experimental pathology, eosinophils are best studied in helminth infection and allergic airway models, yet these settings do not fully capture eosinophil behavior in sterile granulomatous inflammation, foreign material reactions, and repair-associated matrix remodeling (Rosenberg et al. 2013). Well-controlled in vivo models that reliably generate and sustain eosinophil-rich lesions are therefore valuable for comparative pathology, allowing direct mapping of lesion architecture and temporal transitions from acute inflammation to encapsulation under defined experimental conditions.

Granulomas are organized immune structures that form in response to persistent antigens, foreign bodies, or chronic infections, serving as a means to contain inflammatory stimuli and facilitate resolution (Shah et al. 2017). In the past 5 decades, macrophages and T cells have been extensively studied as the primary drivers of the formation of the antigen-specific granuloma (also termed “hypersensitivity granuloma” or “immune granuloma”). However, recent evidence suggests that eosinophils can also play a crucial role in orchestrating granulomatous inflammation, particularly in fibrotic and allergic settings (Rumbley et al. 1999; Bohrer et al. 2021; Malta et al. 2022). However, the mechanisms underlying eosinophil recruitment, persistence, and function within granulomas remain poorly understood. Developing models that capture these dynamics is essential to elucidate eosinophil-mediated immune responses and their potential contributions to tissue remodeling and fibrosis.

The heat-coagulated egg white pellet (EWIp) model (Vieira et al. 2021) provides a unique opportunity to investigate eosinophil dynamics in a controlled, sterile inflammatory environment. This model induces a strong eosinophilic inflammatory response, with a sustained eosinophilic infiltrate. The EWIp model shares important features with both hypersensitivity granulomas (antigen specificity and high turnover) and foreign body-induced granulomas (persistence of the solid material in the tissue), allowing us to explore the interactions of neutrophils with other immune cell types and with the extracellular matrix. This model provides histopathological parallels to eosinophil-rich granulomatous inflammation observed in human fibrotic or foreign-body responses.

Recent findings have highlighted the role of glucocorticoid signaling and 5-lipoxygenase (5-LO) activity in regulating eosinophil recruitment and persistence in inflammation (Vieira et al. 2025). The EWIp model demonstrates that eosinophilia depends on both pathways, raising important questions about how eosinophils contribute to granulomatous inflammation and its resolution.

In this context, the present study aimed to characterize the histological evolution of the eosinophil-rich granuloma induced by a heat-coagulated egg white pellet, focusing on the spatial distribution of inflammatory infiltrates and extracellular matrix components within the pellet–tissue interface, necrotic areas, and outer capsule from day 1 to day 14 after surgery. We evaluated whether the eosinophil-rich phase temporally coincides with organized matrix remodeling and encapsulation in a sterile implant granuloma. The primary aim of this study was to establish an atlas-style, time-resolved histopathological framework of EWIp-induced sterile granuloma maturation, rather than to define cell-specific mechanisms or activation states.

## Methods

### Animals and ethical aspects

Male or female mice 6–8 weeks old of the BP-2 strain were bred at ICTB−FIOCRUZ (Rio de Janeiro, Brazil). The study followed institutionally approved guidelines (CEUA No. L-010/04 and CEUA No. L-002/09, CEUA − CCS − UFRJ, Rio de Janeiro, Brazil). All studies involving animals were reported according to the ARRIVE guidelines for reporting experiments involving animals (McGrath et al. 2010; Kilkenny et al. 2010). Forty (5/day) specific pathogen-free mice were used in the experiments described here. Animals were maintained in microisolator units (a maximum of 5 mice per cage) under a 12 h light/dark cycle at 23 °C and received autoclaved mouse chow and water ad libitum. Euthanasia was performed under an excess CO_2_ atmosphere in a dedicated chamber (Beira-Mar, Rio de Janeiro, Brazil), and all efforts were made to minimize suffering.

### In vivo procedures

#### EWIp

Mice were anesthetized with ketamine (100 mg/kg; Syntec, São Paulo, Brazil) and xylazine (12 mg/kg; Syntec, São Paulo, Brazil), i.p. After local asepsis inside a hood, an approximately 1-cm access to the peritoneal cavity was gained, and a sterile pellet of heat-coagulated egg white (Ito Avicultura, São Paulo, Brazil) was inserted with tweezers, before suturing together tissue layers from skin to peritoneum. With their eyes protected, mice recovered from anesthesia under a 40-W lamp.

### Ex vivo procedures

#### Harvest and quantification of leukocyte populations

After euthanasia, the peritoneal cavity was exposed under aseptic conditions, and peritoneal exudate cells (PEC) were collected by lavage with 10 mL of RPMI 1640 medium (Thermo Fisher, Waltham, MA, USA) containing 25 U/mL heparin. The lavage fluid was gently injected into the peritoneal cavity, recovered after gentle abdominal massage, and transferred to sterile tubes kept on ice. A total of 9 mL of recovered lavage fluid was used for leukocyte quantification and cytological analysis. PEC suspensions were centrifuged at 500 × *g*  for 15 min at 4 °C, the supernatant was discarded, and the cell pellet was resuspended in 2 mL of RPMI 1640 medium.

Total leukocyte numbers were determined in a hemocytometer after dilution in Turk’s solution. Differential leukocyte counts were performed on cytocentrifuge preparations. Briefly, aliquots of PEC suspensions were deposited onto glass slides by cytocentrifugation (500 × *g*  for 5 min), air-dried, fixed in formalin vapor for 15 min, and stained for eosinophil peroxidase (EPX), followed by hematoxylin counterstaining. Eosinophils were identified by EPX-positive cytoplasmic staining and characteristic granulocyte morphology. Neutrophils and mononuclear phagocytes were identified according to standard cytomorphological criteria. At least 300 cells were counted per cytocentrifuge smear. The relative frequency of each leukocyte population was determined from differential counts, and absolute numbers of eosinophils, neutrophils, and mononuclear phagocytes were calculated by multiplying the total leukocyte count by the corresponding differential percentage.

### Histology

After collection of the peritoneal exudate, the egg white implant together with the surrounding omental tissue was carefully excised to preserve the spatial relationship between the pellet, pellet–tissue interface, and adjacent capsule/omentum. Tissue samples were fixed in Carson’s formalin (Millonig) for 48 h at room temperature (Carson et al. 1973) and then processed according to standard histological procedures for paraffin embedding. Paraffin blocks were sectioned at 5 µm thickness, and serial sections were mounted on glass slides for routine and special histochemical staining.

Sections were stained with hematoxylin and eosin (H&E) for general tissue architecture, inflammatory infiltrate composition, necrosis/tissue degeneration, apoptotic bodies, fibroblast-rich areas, vascular profiles, and granuloma organization. Masson’s trichrome staining was used to evaluate fibrinous/exudative material and collagen deposition during capsule formation. Gomori’s reticulin staining was used to assess reticular fiber organization within the granuloma and capsule. Picrosirius staining was used to evaluate collagen-rich extracellular matrix deposition. Sirius Red staining at pH 10.2 was used to highlight eosinophils within the inflammatory infiltrate and at the pellet–tissue interface.

Stained slides were examined by bright-field light microscopy using an Axiovert Z1 microscope (Carl Zeiss, Oberkochen, Germany) equipped with ×10, ×20, ×40, and ×100 objective lenses and an mRC5 Axiocam digital camera (Carl Zeiss, Oberkochen, Germany). Digital images were acquired using ZEN Blue software, version 3.8.99.01000 (Carl Zeiss, Oberkochen, Germany), under standard bright-field illumination conditions. Representative images were selected to document predefined lesion compartments, including the pellet–tissue interface, inflammatory infiltrate, necrotic or degenerative areas, outer capsule, adjacent omental tissue, and extracellular matrix organization. Images were exported from the original microscopy files as high-resolution digital images for figure preparation. Only global adjustments of brightness, contrast, and color balance, when necessary, were applied uniformly to the entire image. No selective enhancement, deletion, relocation, or modification of histological structures was performed.

### Histopathology scoring

To summarize temporal changes in lesion composition, slides were evaluated compartment-by-compartment (pellet–tissue interface and outer capsule) across the day 1–14 time course. For each animal, stain, and time point, one pathologist captured images from three non-overlapping representative fields (400 ×) per compartment. Fields were selected within predefined compartments (pellet–tissue interface and outer capsule) to avoid tissue folds and section edges. A second pathologist, blinded to group/time point, independently assigned ordinal scores for each field. Semiquantitative scoring was operationalized using predefined, field-based criteria to reduce subjectivity and improve reproducibility. For (i) macrophage/mononuclear phagocyte–rich infiltration (H&E), scores were assigned as 0 when cells were absent or extremely rare (0–1 cell/HPF), 1 when there was a sparse, non-confluent infiltrate occupying approximately < 25% of the field, 2 when infiltration was frequent/multifocal and involved approximately 25–50% of the field, and 3 when a dense or confluent infiltrate involved approximately > 50% of the field; (ii) eosinophil-rich infiltration (Sirius Red pH10.2) was scored using a primary quantitative criterion: 0 for 0–1 eosinophils/HPF, 1 for 2–10/HPF, 2 for 11–30/HPF, and 3 for > 30/HPF or a dense, band-like accumulation at the pellet–tissue interface; (iii) fibrinous exudate and fibrin networks (Masson’s trichrome) were scored on the designated fibrin-sensitive stain as 0 when absent, 1 when present as thin/discontinuous strands occupying approximately < 25% of the field, 2 when readily apparent networks occupied approximately 25–50% of the field, and 3 when extensive, dominant fibrin networks involved approximately > 50% of the field; (iv) fibroplasia/collagen deposition and capsule maturation (Masson’s trichrome) were scored as 0 when absent, 1 for early fibroblast activation with sparse collagen and a discontinuous capsule, 2 for a clearly defined capsule of moderate thickness with partially organized collagen bundles, and 3 for a thick, continuous, mature collagenous capsule with dense, well-organized bundles; (v) reticulin fiber organization (Gomori’s reticulin) was scored as 0 when absent, 1 when fibers were sparse and disorganized, 2 when a moderate, partially organized reticulin framework was evident, and 3 when a dense, well-organized reticulin framework delineated lesion architecture and/or the capsule; (vi) necrosis/necroptotic tissue degeneration and apoptotic bodies (H&E) were scored as 0 when absent with minimal debris, 1 for small focal areas of degeneration with limited debris and occasional apoptotic bodies, 2 for a clearly defined degenerative/necrotic zone with abundant debris and frequent apoptotic bodies, and 3 for extensive, confluent necrosis/necroptotic change dominating the compartment with widespread debris and numerous apoptotic bodies; (vii) neovascularization (H&E) was scored as 0 when absent, 1 when rare newly formed vessels were present (approximately 1–2/HPF), 2 when vascular profiles were clearly increased (approximately 3–5/HPF), and 3 when prominent vascularization was present (> 5/HPF) or showed a diffuse distribution across the compartment.

For each animal, field-level scores were averaged across the three fields to obtain a per-animal score for each feature and time point. Group values were then calculated as the median per-animal score (*n* = 5 animals/time point). A composite remodeling score was computed per animal and then summarized per day; because fibrin is expected to decline as remodeling progresses, its daily value was transformed as (3 − fibrin score) before summation.

### Data Analysis

Data were analyzed using SigmaPlot for Windows (version 11.0; Grafiti LLC, Palo Alto, CA, USA). Cytological counts are presented as mean ± SEM. For cytology outcomes, differences across time points were assessed using a one-way analysis of variance (ANOVA) with a Bonferroni correction for multiple comparisons; when the assumptions of normality or equal variance were not met, the Kruskal–Wallis test, followed by Dunn’s post hoc test, was used instead.

#### Semiquantitative histopathology

Scores (0–3) were treated as ordinal outcomes and are presented as median [IQR] (supplementary methods). For each feature, differences across time points (days 1, 2, 3, 4, 5, 6, 7, and 14; *n* = 5 animals/time point) were evaluated using the Kruskal–Wallis test, followed by Dunn’s multiple-comparison post hoc test with Holm adjustment. Monotonic temporal progression across ordered time points was assessed using the Jonckheere–Terpstra trend test. For the fibrinous exudate/network component, which is expected to decline during remodeling, directionality was addressed by inverting daily values (3 − fibrin score) before incorporating them into the composite remodeling score. A composite daily remodeling score was computed as the sum of oriented feature scores for each day after this step. Two-sided tests were used, and *p* < 0.05 was considered statistically significant.

#### Association analyses (eosinophil score vs remodeling features)

To evaluate whether eosinophil enrichment was associated with changes in lesion composition, we assessed rank correlations between the eosinophil-rich infiltration score and key remodeling features. Because multiple features progress over time and may co-vary with “day”, we performed (i) unadjusted Spearman rank correlations using per-animal values pooled across time points and (ii) partial rank correlations controlling for day as an ordered covariate (rank-based residual approach) to evaluate associations beyond temporal progression. Correlation coefficients (ρ) and two-sided* p* values are reported (Supplementary Tables S1, S2).

## Results

### Day 1

**Fig. 1:**
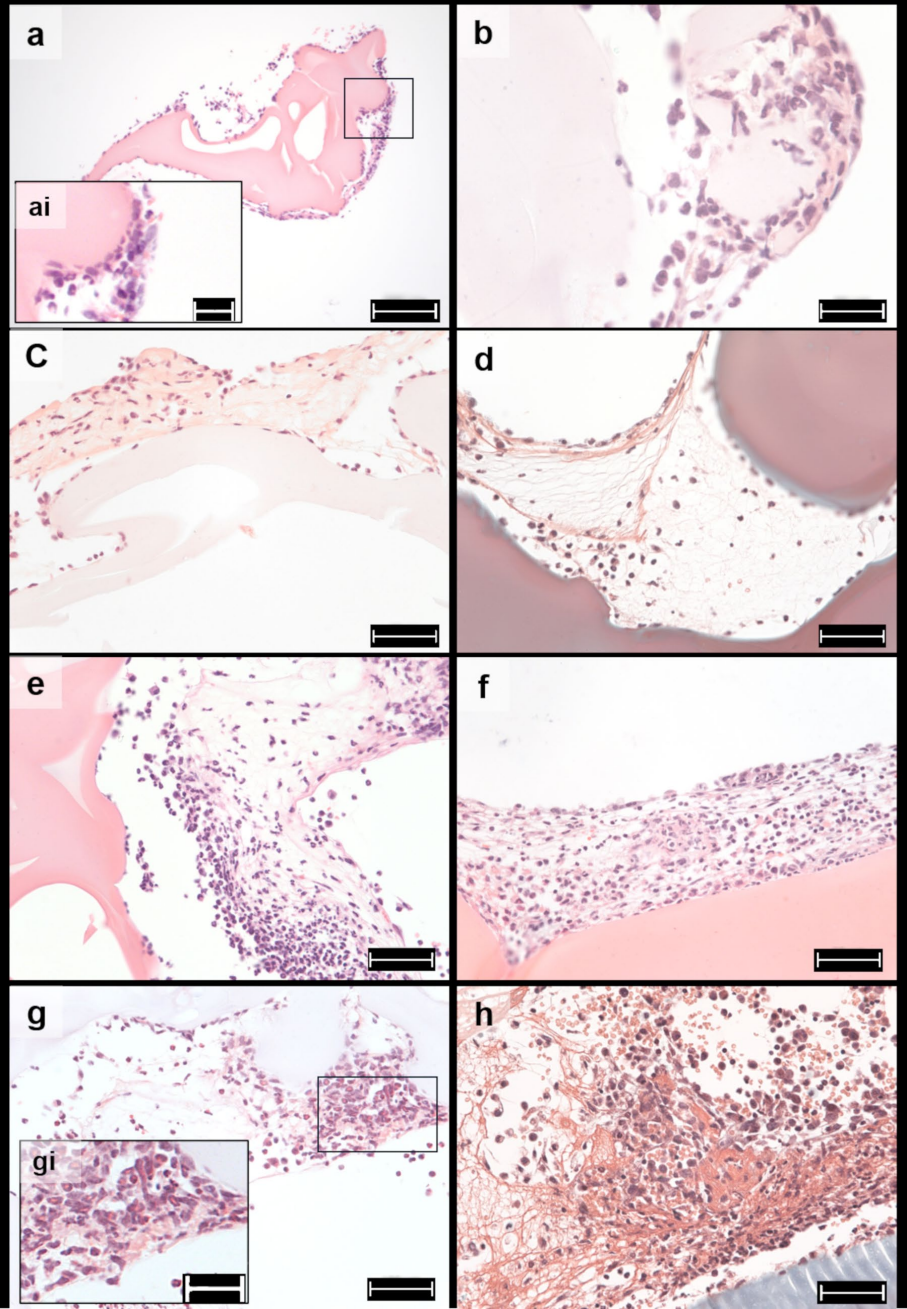
Early inflammatory profile and matrix organization on days 1 and 2. **a**–**d** Histological sections from day 1 post-implantation. **a**, **b** H&E: limited neutrophilic and eosinophilic infiltration near the pellet. **ai** Inset of the boxed region in panel** a**. **c** Sirius Red: early identification of scattered eosinophils in the inflammatory infiltrate. **d** Masson’s trichrome: absence of organized collagen deposition around the pellet. **e**–**h** Histological sections from day 2. **e** H&E: increased inflammatory infiltration with greater eosinophil and mononuclear phagocyte presence. **f** Sirius Red: evident eosinophilic degranulation and apoptotic bodies. **g** Sirius Red: elevated number of eosinophils surrounding the pellet.** gi** Inset of the boxed region in panel** g**. **h** Masson’s trichrome: disorganized extracellular matrix without structured fibroplasia. Scale bars: **a** = 100 μm; **ai** = 20 μm; **b** = 20 μm; **c** = 50 μm; **d** = 50 μm; **e** = 100 μm; **f** = 50 μm; **g** = 100 μm;** gi** = 50 μm; **h** = 50 μm

On the first day, a modest inflammatory infiltrate predominantly composed of neutrophils is observed around the pellet, along with fewer eosinophils and macrophages closely associated with the pellet material (H&E—Fig. 1a, ai; Supplementary 1a, b. Sirius Red—Fig. 1b, c). Apoptotic bodies are also in the inflammatory infiltrate (Supplementary 1a, b). Minimal deposition of non-collagenous matrix material is linked to the inflammatory response, and fibrin networks can be observed surrounding the pellet, as seen in Sirius Red (Fig. 1c; Supplementary 1c) and Masson’s trichrome staining (Fig. 1d; Supplementary 1d). Non-collagenous matrix material appears in blue hues, and no collagen fiber formation was seen (Fig. 1d). No reticular fibers are detected (Gomori’s reticulin—not shown).

### Day 2

By the second day, there is an increase in cellularity around the pellet, with a slight rise in eosinophil numbers and an abundance of macrophages and other mononuclear cells, indicating an evolving inflammatory response (H&E—Fig. 1e; Supplementary 1e. Sirius Red—Fig. 1f, g; Supplementary 1f, g). Eosinophils undergoing apoptosis, with pyknotic nuclei closely associated with the pellet, remain evident (Sirius Red—Fig. 1g, gi; Supplementary 1f, g). Apoptotic bodies remain within the inflammatory infiltrate. No evident fibroplasia is observed. Masson’s trichrome staining highlights a primarily non-collagenous matrix surrounding the pellet, with no signs of collagen fiber organization (Fig. 1h; Supplementary 1 h).

### Day 3

**Fig. 2:**
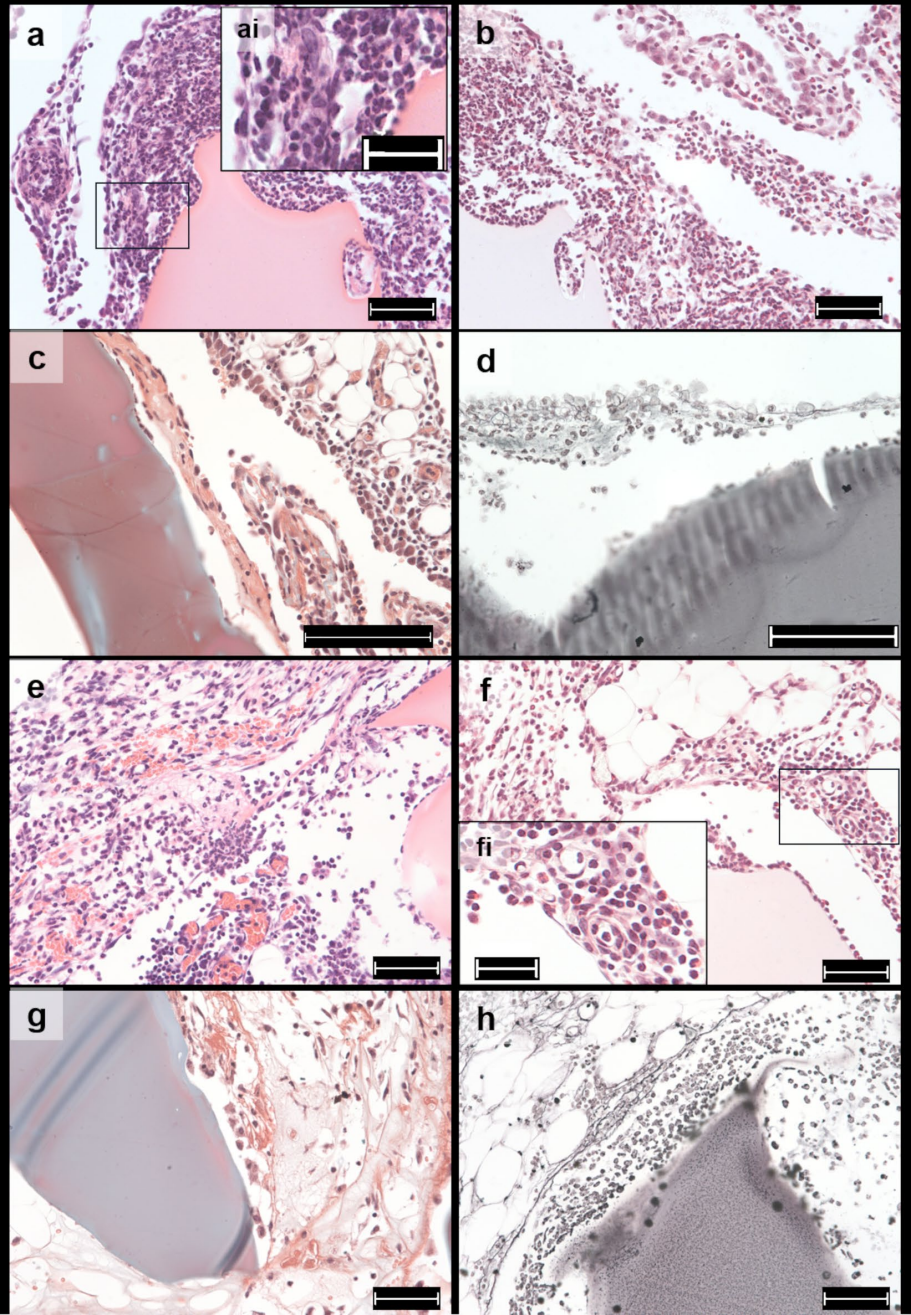
Eosinophilic dominance and early granuloma organization on days 3 and 4. **a**–**d** Histological sections from day 3. **a** H&E: intense eosinophilic infiltration and early fibroplasia.** ai **Inset of the boxed region in panel **a**. **b** Sirius Red: Numerous eosinophils concentrated around the pellet and within the matrix. **c** Masson’s trichrome: initial signs of collagen deposition in the outer capsule. **d** Gomori’s reticulin: thin reticular fibers emerging in the periphery of the granuloma. **e**–**h** Histological sections from day 4. **e** H&E: dense eosinophilic accumulation, apoptotic bodies, and macrophages near the implant. **f** Sirius Red: persistent eosinophil dominance throughout the granuloma structure. (fi) Inset of the boxed region in panel** f**. **g** Masson’s trichrome: thickened blue-stained collagen matrix outlining the granuloma. **h** Gomori’s reticulin: increased fiber organization defining the nascent granuloma capsule. Scale bars: **a** = 50 μm; **ai** = 20 μm; **b** = 50 μm; **c** = 100 μm; **d** = 100 μm; **e** = 50 μm; **f** = 50 μm;** fi** = 20 μm; **g** = 50 μm; **h** = 50 μm

On the third day, the inflammatory infiltrate becomes more intense and predominantly eosinophilic, with almost no neutrophils observed (H&E—Fig. 2a; Supplementary 2a. Sirius Red—Fig. 2b; Supplementary 2c). There is an increase in macrophages and rare multinucleated giant cells, indicating a progressing immune response (Fig. 2a, ai; Supplementary 2b). Signs of vascular formation and leakage are evident, with increased eosinophil infiltration around newly formed blood vessels. Fibroplasia becomes apparent, with increasing collagen deposition (Fig. 2c; Supplementary 2d) and reticular fibers (Fig. 2d). In H&E staining, eosinophils with cytoplasmic granules remain concentrated around the pellet, indicative of localized inflammatory activation. Granuloma formation appears to be underway, contributing to the early encapsulation of the pellet (Fig. 2a; Supplementary 2b).

### Day 4

By the fourth day, the area around the pellet contains numerous eosinophils, many of which are undergoing degranulation, particularly near the pellet (H&E—Fig. 2e; Supplementary 2e. Sirius Red—Fig. 2f, fi; Supplementary 2f, 2 g). This process is associated with reduced matrix deposition and gradual distancing of the pellet from the inflammatory infiltrate. The pellet shows evident signs of degeneration, with the emergence of trabecular structures. Neovascularization is prominent, corroborating remodeling of the local vascular environment (H&E, Fig. 2e). Fibroblasts become reactive, corroborating active fibroplasia, and increased collagen deposition is associated with this process (Fig. 2g). A granulomatous structure becomes more evident, with a developing capsular organization composed of connective tissue and inflammatory cells surrounding the pellet.

Masson’s trichrome and Gomori’s reticulin staining (Masson’s—Fig. 2g; Gomori’s—Fig. 2h; Supplementary 2h) reveals an increase in both collagen and reticular fiber organization, reinforcing the structural complexity of the granuloma. Smaller pellet fragments are progressively engulfed, while larger structures remain more exposed, continuing to attract eosinophilic infiltration and neovascularization. The encapsulating structure becomes more intricate, with connective tissue and inflammatory components forming a more defined barrier around the pellet.

### Day 5

**Fig. 3:**
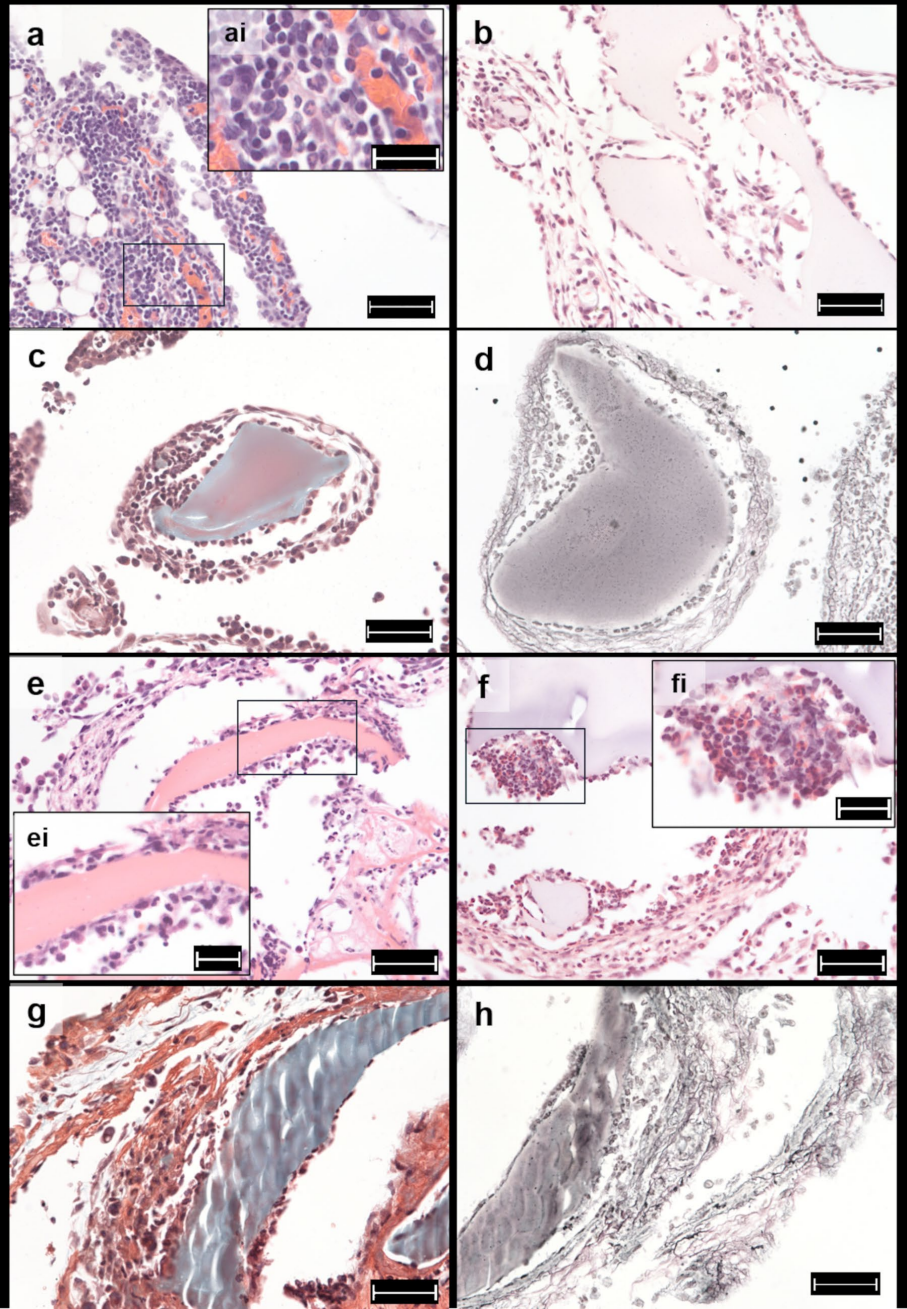
Granuloma maturation and matrix remodeling on days 5 and 6. **a**–**d** Histological sections from day 5. **a** H&E: encapsulated granuloma with eosinophils infiltrating both capsule and internal trabeculae. **ai** Inset of the boxed region in panel **a**. **b** Sirius Red: eosinophilic zones around the pellet and within granuloma trabeculae. **c** Masson’s trichrome: collagen deposition forming a multilayered pericapsular scaffold. **d** Gomori’s reticulin: reticular fiber alignment along the capsule and trabeculae. **e**–**h** Histological sections from day 6. **e** H&E: formation of necrotic areas rich in eosinophils and apoptotic debris. **ei** Inset of the boxed region in panel **e**. **f** Sirius Red: widespread eosinophil infiltration and fibrin-rich matrix.** fi** Inset of the boxed region in panel **f**. **g** Masson’s trichrome: collagen accumulation at the granuloma periphery. **h** Gomori’s reticulin: an organized fiber network in mononuclear areas, absent in polymorphonuclear zones. Scale bars: **a** = 50 μm; **ai** = 20 μm; **b** = 50 μm; **c** = 50 μm; **d** = 50 μm; **e** = 50 μm; **ei** = 20 μm; **f** = 50 μm; **fi** = 20 μm; **g** = 50 μm; **h** = 50 μm

On the fifth day, the pellet is surrounded by a more defined capsule, further consolidating its structural integrity and isolating the pellet. The degenerative process of the pellet mass continues, with inflammatory infiltration advancing into the trabeculae formed by its degradation (Fig. 3b). An intense eosinophilic infiltrate is observed. Surrounding milky spots exhibit intense activation, accompanied by significant vascularization and a high density of eosinophils within these structures (Fig. 3a, ai).

Sirius Red staining reveals a few apoptotic eosinophils with pyknotic nuclei near the capsule, suggesting active cellular turnover (Supplementary 3a, 3b). Collagen fibers are more prominently deposited in the distal region, with Masson’s trichrome staining (Fig. 3c; Supplementary 3c) showing blue hues of collagen fibers forming a capsule around the pellet, along with reticular fibers (Fig. 3d; Supplementary 3d), corroborating the development of a fibrous layer that further defines the encapsulation, particularly along the outer edge of the capsule, reflecting ongoing matrix maturation. The fibrin network is notably reduced, reinforcing the transition toward a more organized granulomatous response.

### Day 6

On the sixth day, the granuloma is well defined, with a noticeable reduction in pellet mass and an eosinophilic infiltrate extending into it. A necrotic region containing polymorphonuclear cells and fibroblasts is evident around the pellet (Fig. 3e; Supplementary 3e). Numerous mononuclear phagocyte/macrophage-like cells, often adjacent to cellular remnants and apoptotic bodies, are present, along with a significant number of apoptotic bodies. Mitotic figures are also observed, suggesting ongoing cellular proliferation within the inflammatory response (Fig. 3f; Supplementary 3e, 3f, and 3g).

Masson’s trichrome staining (Fig. 3g; Supplementary 3h) reveals fibrillar connective tissue degeneration interspersed among inflammatory cells at the periphery of the pellet, appearing in red. There is a significant deposition of collagen in inflammatory regions, particularly in areas dominated by mononuclear cells. H&E and Sirius Red staining (Fig. 3e, ei, f, fi) highlight the eosinophilic infiltrate surrounding and infiltrating the pellet, with apoptotic eosinophils indicating continued immune activity and cellular turnover. While no reticular fibers are deposited among the polymorphonuclear inflammatory cells, a well-organized reticular fiber network is observed in surrounding areas where mononuclear cells predominate (Fig. 3h). A moderate division into three distinct zones around the pellet is emerging, reflecting an advanced stage of granuloma organization.

### Day 7

**Fig. 4:**
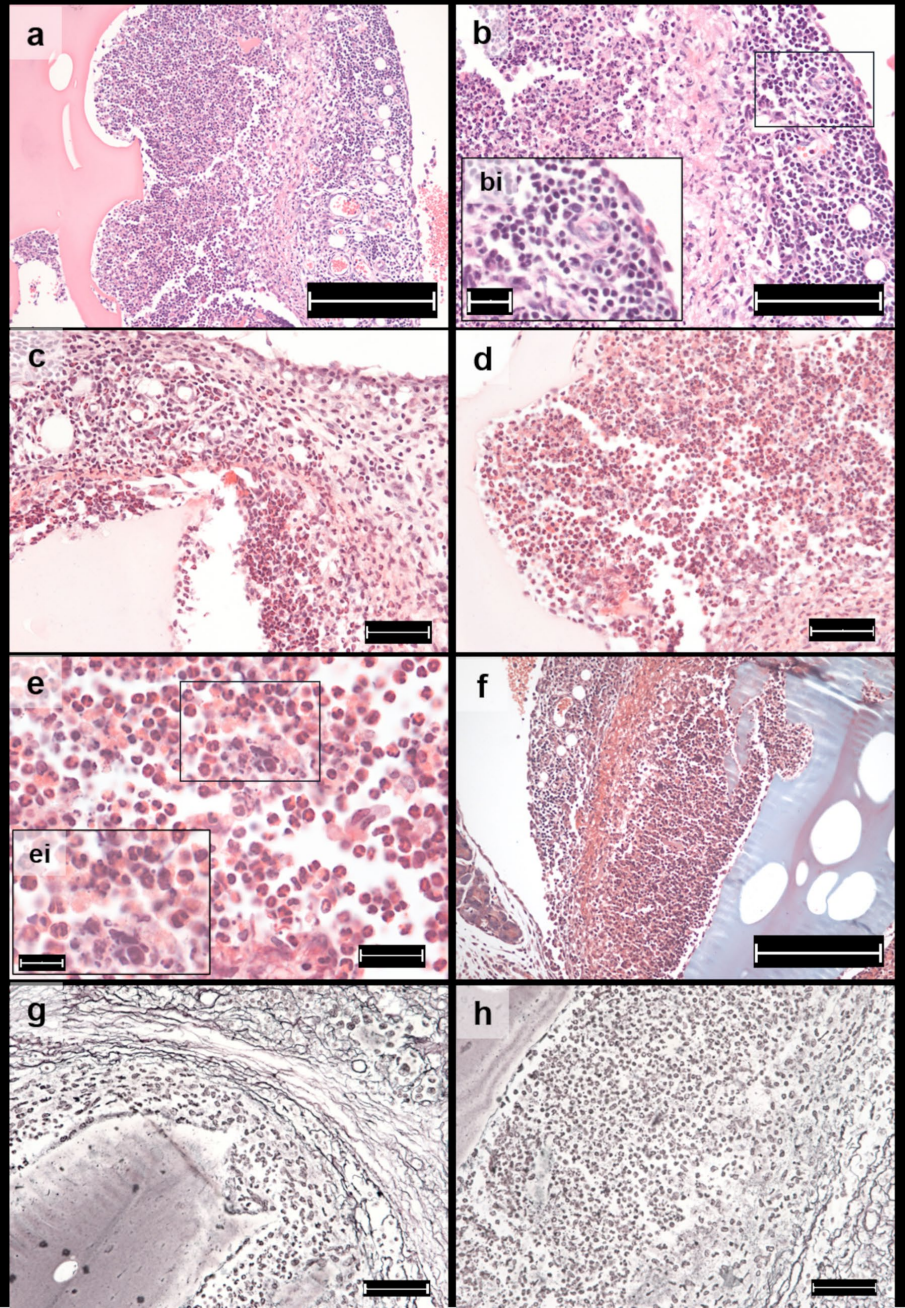
Peak eosinophilic activity and structured granuloma zones on day 7. **a**–**e** Histological sections from day 7. **a**, **b** H&E: full granuloma structure with zonal distribution—central eosinophilic core, necrotic middle zone, and lymphocytic/fibroblastic outer capsule.** bi** Inset of the boxed region in panel **b**. **c**–**e** Sirius Red: intense eosinophilic degranulation, apoptotic eosinophils, and immune cell accumulation around the pellet and within the necroptotic zone.** ei** Inset of the boxed region in panel **e**. **f** Masson’s trichrome: collagen deposition outlining granuloma and adjacent tissue. **g**, **h** Gomori’s reticulin: reticular fiber presence in the capsule; absent from necrotic zones. Scale bars: **a** = 200 μm; **b** = 100 μm;** bi** = 20 μm; **c** = 50 μm; **d** = 50 μm; **e** = 20 μm; **ei** = 10 μm; **f** = 200 μm; **g** = 50 μm; **h** = 50 μm

By the seventh day, the granuloma is fully encapsulated by a well-organized matrix and inflammatory cells, with three distinct zones becoming more evident (Fig. 4a, b). The innermost zone consists of a dense layer of polymorphonuclear cells, predominantly eosinophils, closely associated with apoptotic bodies around the pellet, where intense eosinophil degranulation is observed (Fig. 4c, d, e, ei). The middle zone is necrotic, exhibiting extensive cellular debris and apoptotic remnants. The outermost zone is rich in lymphocytes, fibroplasia, and vascularization, indicating an advanced stage of tissue remodeling (Fig. 4a–e). Reactive fibroblasts are prominent, and the entire structure appears to be enclosed within a layer suggestive of omentalization (Fig. 4a, b, bi).

Macrophages with an epithelioid morphology are seen in association with vascularization and fibroplasia, suggesting the early stages of tissue repair (Fig. 4a, b, bi). Masson’s trichrome staining (Fig. 4f) highlights connective tissue and collagen deposition within the necrotic zone. In contrast, Gomori’s reticulin (Fig. 4g, h) staining does not show positivity in this area, indicating fibrin presence. In the outer zone, there is significant deposition of collagen and reticular fibers, reinforcing the granuloma’s structural maturation (Fig. 4f–h). Vascularization forms a rounded pattern around the pellet, resembling a mature granuloma. Giant cells appear between the proximal and distal zones, possibly resulting from macrophage fusion in response to the foreign material.

### Day 14

**Fig. 5:**
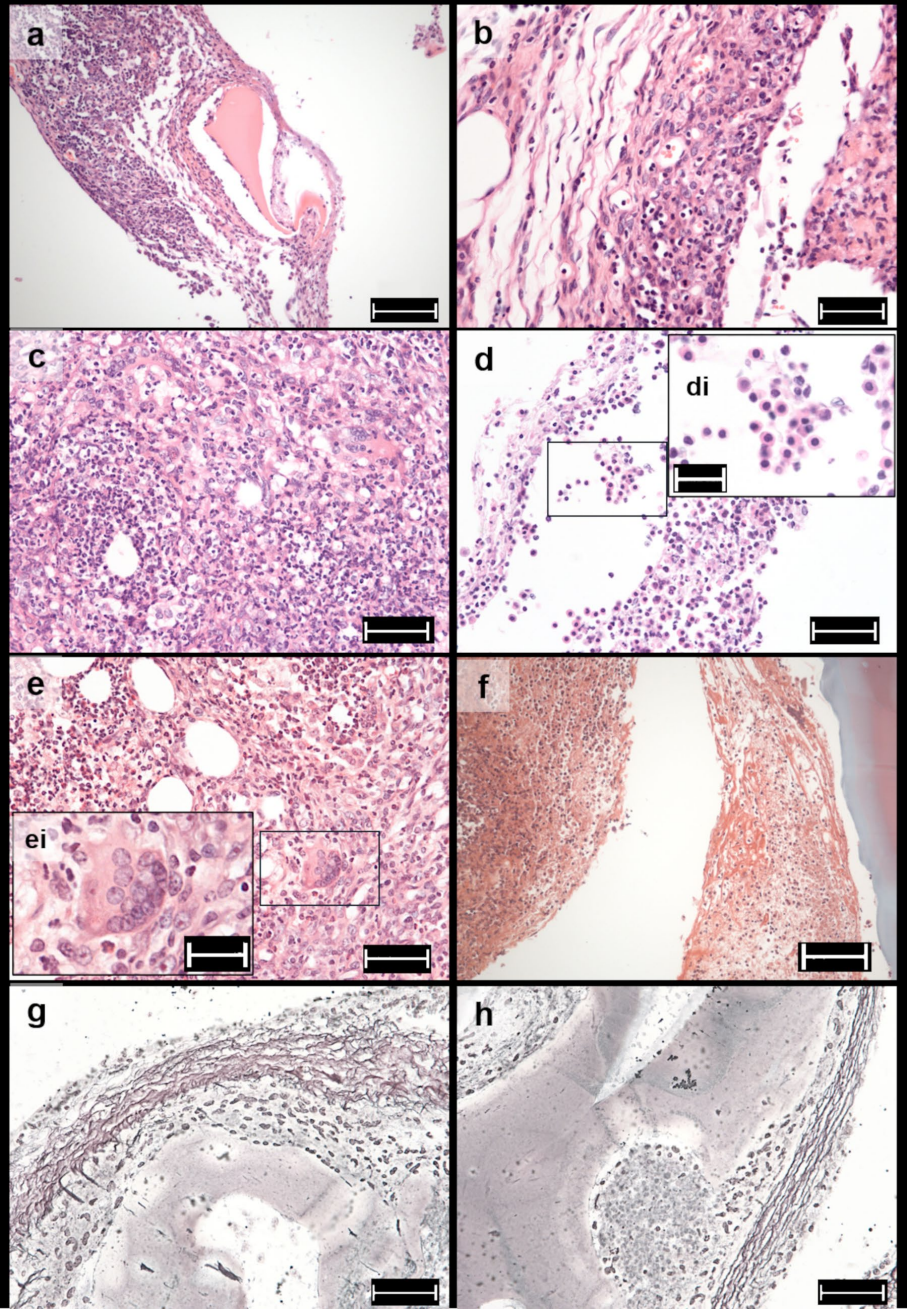
Mature granuloma with tissue remodeling on day 14 (**a**–**e**). Histological sections from day 14. **a**,** b** H&E: encapsulated granuloma with collapsed necrotic core and scattered eosinophilic infiltrate. **c**–**e** Sirius Red: eosinophils remain present within trabeculae and pericapsular zones; apoptotic bodies and fibroblasts are identified. **di** Inset of the boxed region in panel** d**. **ei** Inset of the boxed region in panel** e**. **f** Masson’s trichrome: dense, blue-stained collagen outlining the mature capsule and omental interface. **g**, **h** Gomori’s reticulin: reticular fiber network in the capsule, indicating mature stromal organization. Scale bars: **a** = 100 μm; **b** = 50 μm; **c** = 50 μm; **d** = 50 μm;** di** = 20 μm; **e** = 50 μm; **ei** = 20 μm; **f** = 100 μm; **g** = 50 μm; **h** = 50 μm

By the 14th day, a fully developed foreign body granuloma with dense connective tissue and apoptotic inflammatory cells is observed (H&E—Fig. 5a). The pellet mass is highly degenerated, transitioning the inflammatory process into a resolution phase. The previously distinct intimate inflammatory zone and necrotic zone have merged into a single necroptotic zone, characterized by an amorphous necrotic mass interspersed with abundant apoptotic bodies. Surrounding the pellet and the necroptotic zone, eosinophils remain predominant, but the infiltrate is less intense compared to earlier stages (Fig. 5b, c, e).

In the outer granuloma zone, mononuclear phagocyte/macrophage-like cells with a relatively homogeneous morphology were observed (Fig. 5d, di). These cells are morphologically distinct from those described earlier. They display a more homogeneous and monotonous appearance, characterized by a slightly peripheral, round nucleus with condensed chromatin, and a lightly eosinophilic, uniform cytoplasm with well-defined boundaries and no cytoplasmic projections. There is a prominent deposition of collagen in the external layers, encapsulating the necroptotic zone (Fig. 5f). Well-organized reticular fibers are observed adjacent to this region, reinforcing the structural maturation of the granuloma (Fig. 5b, f). The co-localization of fibrin and collagen, as evidenced by Masson’s trichrome (Fig. 5f), suggests a transitional stage between necrosis and tissue repair.

In the adjacent omentum, the inflammatory process persists, characterized by a classical granuloma. Gomori’s reticulin (Fig. 5g, h) staining highlights an intricate reticular fiber network permeating the granuloma, with prominent mitotic figures, multinucleated giant cells, reactive and dysplastic fibroblasts, and epithelioid macrophages (Fig. 5c, e). Eosinophils are interspersed among other inflammatory cells within the omentum.

### Macroscopy and neutrophil/eosinophil dynamics

**Fig. 6:**
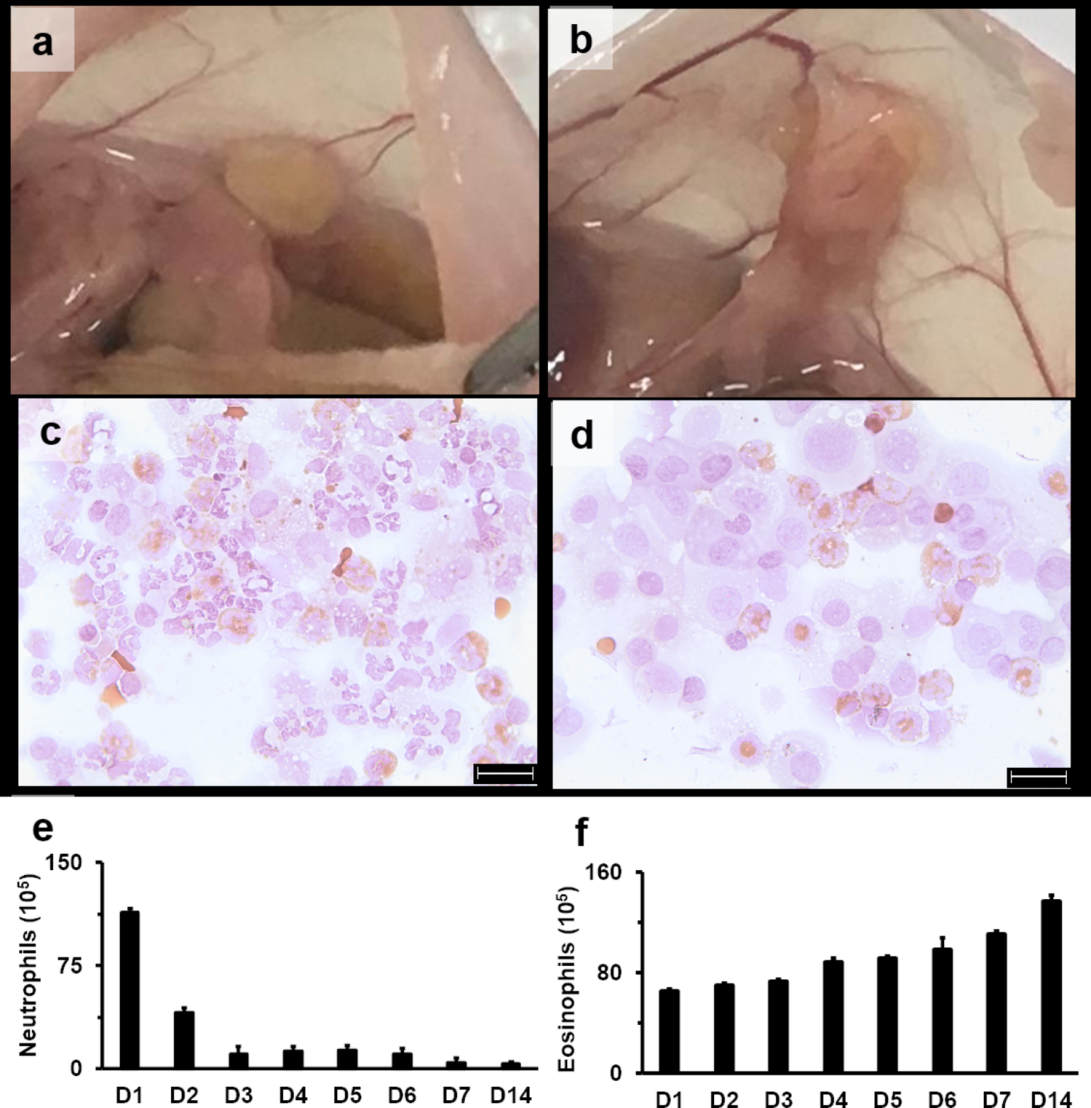
Macroscopic encapsulation and cellular dynamics of granulocyte adsorption. **a** Day 1: macroscopic view showing the pellet freely suspended in the peritoneal cavity. **b** Day 14: the pellet is completely encapsulated by omental tissue. **c** Cytospin on day 1: predominance of neutrophils among adherent cells. **d** Cytospin on day 14: predominance of eosinophils. **e** Quantification: decline in neutrophils in peritoneal exudate over time. **f** Quantification: progressive increase in eosinophil numbers from day 1 to day 14. Scale bars: **c** = 50 μm; **d** = 50 μm

Upon macroscopic examination, the histologically described encapsulation process was readily apparent. Figure 6a illustrates the pellet on day 1 post-surgery, showing minimal external coverage. In contrast, Fig. 6b shows the pellet on day 14 post-surgery with evident omentum wrapping (omentalization) and complete enclosure by connective tissue.

To assess leukocyte dynamics in the peritoneal exudate, cytological counts were performed across the day 1–14 time course (*n* = 5 animals/time point). Neutrophil counts (× 10^5^) were highest at day 1 (113.5 ± 2.5) and declined sharply thereafter (day 2, 41.1 ± 3.1; day 3, 10.9 ± 4.8), reaching low levels by day 14 (3.7 ± 0.6) (Fig. 6e). In contrast, eosinophil counts (× 10^5^) increased over time, rising from day 1 (74.0 ± 4.1) through day 6 (319.5 ± 24.5) and peaking at day 14 (540.0 ± 74.4) (Fig. 6f). Together, these cytological data support a transition from an early neutrophil-predominant response to a late eosinophil-enriched phase during lesion maturation.

### Histopathological score

Semiquantitative ordinal scoring (0–3) across independent time points (*n* = 5 animals/day) showed significant differences among days for all lesion components (Kruskal–Wallis, all *p* ≤ 1.4 × 10^−5^). Trend analysis across ordered time points confirmed a strong monotonic increase for all remodeling-related features (Jonckheere–Terpstra, all *p* < 0.001), whereas fibrinous exudate/networks showed a significant monotonic decline over time (Jonckheere–Terpstra, *p* < 0.001). Median [IQR] values are summarized in Table 1.

**Table 1:** Semiquantitative histopathology scoring across the EWIp time course (days 1–14)

	Day								Kruskal–Wallis* p*	Trend (Spearman ρ),* p*
	1	2	3	4	5	6	7	14		
Mononuclear phagocyte-rich infiltration	0.00 [0.00–0.00]	0.33 [0.33–0.67]	1.33 [1.00–2.00]	2.00 [2.00–2.00]	2.00 [2.00–2.00]	1.67 [1.00–2.00]	2.33 [2.00–3.00]	3.00 [3.00–3.00]	1.05e−05	0.868, 4.09e−13
Eosinophilic infiltration	0.00 [0.00–0.33]	1.00 [1.00–1.33]	1.00 [1.00–1.00]	1.33 [1.33–2.00]	2.00 [1.67–2.00]	2.67 [2.67–3.00]	2.67 [2.67–3.00]	3.00 [3.00–3.00]	6.24e−06	0.942, 1.41e−19
Fibrinous exudate/networks	3.00 [3.00–3.00]	3.00 [2.67–3.00]	2.33 [2.33–2.67]	2.33 [2.33–2.33]	1.67 [1.67–1.67]	1.33 [1.33–2.33]	1.33 [1.33–1.33]	0.33 [0.33–0.67]	9.53e−06	-0.940, 2.42e−19
Fibroplasia/collagen deposition & capsule maturation	0.00 [0.00–0.00]	0.33 [0.00–0.33]	0.33 [0.33–0.33]	0.67 [0.33–0.67]	1.00 [0.67–1.67]	1.00 [1.00–1.33]	2.00 [2.00–2.00]	2.67 [2.67–3.00]	1.14e−05	0.938, 5.07e−19
Reticulin fiber organization	0.00 [0.00–0.00]	0.00 [0.00–0.00]	1.00 [0.67–1.00]	2.00 [2.00–2.33]	2.00 [2.00–2.33]	2.33 [2.00–2.67]	3.00 [2.00–3.00]	3.00 [3.00–3.00]	1.37e−05	0.918, 8.24e−17
Necrosis, tissue degeneration & apoptotic bodies	0.00 [0.00–0.00]	0.33 [0.33–0.33]	0.67 [0.67–0.67]	1.33 [1.00–1.33]	2.00 [1.67–2.00]	2.33 [2.33–2.67]	3.00 [3.00–3.00]	3.00 [3.00–3.00]	5.86e−06	0.964, 2.17e−23
Neovascularization	0.00 [0.00–0.00]	0.00 [0.00–0.00]	0.67 [0.00–1.00]	2.00 [2.00–2.00]	2.00 [2.00–2.00]	2.00 [1.67–2.00]	2.00 [2.00–2.33]	3.00 [3.00–3.00]	6.50e−06	0.901, 2.46e−15
Total	0.33 [0.00–0.33]	2.32 [2.00–2.33]	5.33 [5.00–6.34]	9.67 [9.66–10.67]	12.33 [11.33–12.34]	13.00 [13.00–13.34]	16.32 [16.01–17.34]	20.33 [20.00–20.34]	2.79e−06	0.989, 5.55e−33

Values are shown as median [IQR] for each feature at each time point (*n* = 5 animals/time point). Scores were assigned on an ordinal 0–3 scale (0 = absent, 1 = mild, 2 = moderate, 3 = marked) from three non-overlapping fields per animal within predefined lesion compartments, and field-level scores were averaged to obtain a per-animal value. The rightmost columns report the overall Kruskal–Wallis *p* value for differences across time points and the Spearman rank correlation coefficient (*ρ*) with its p value for temporal trend across ordered days. “TOTAL” represents the composite daily remodeling score computed per animal as the sum of oriented feature scores, with the fibrinous exudate/networks component inverted (3 − fibrin score) before summation to reflect its expected decline during remodeling

**Fig. 7:**
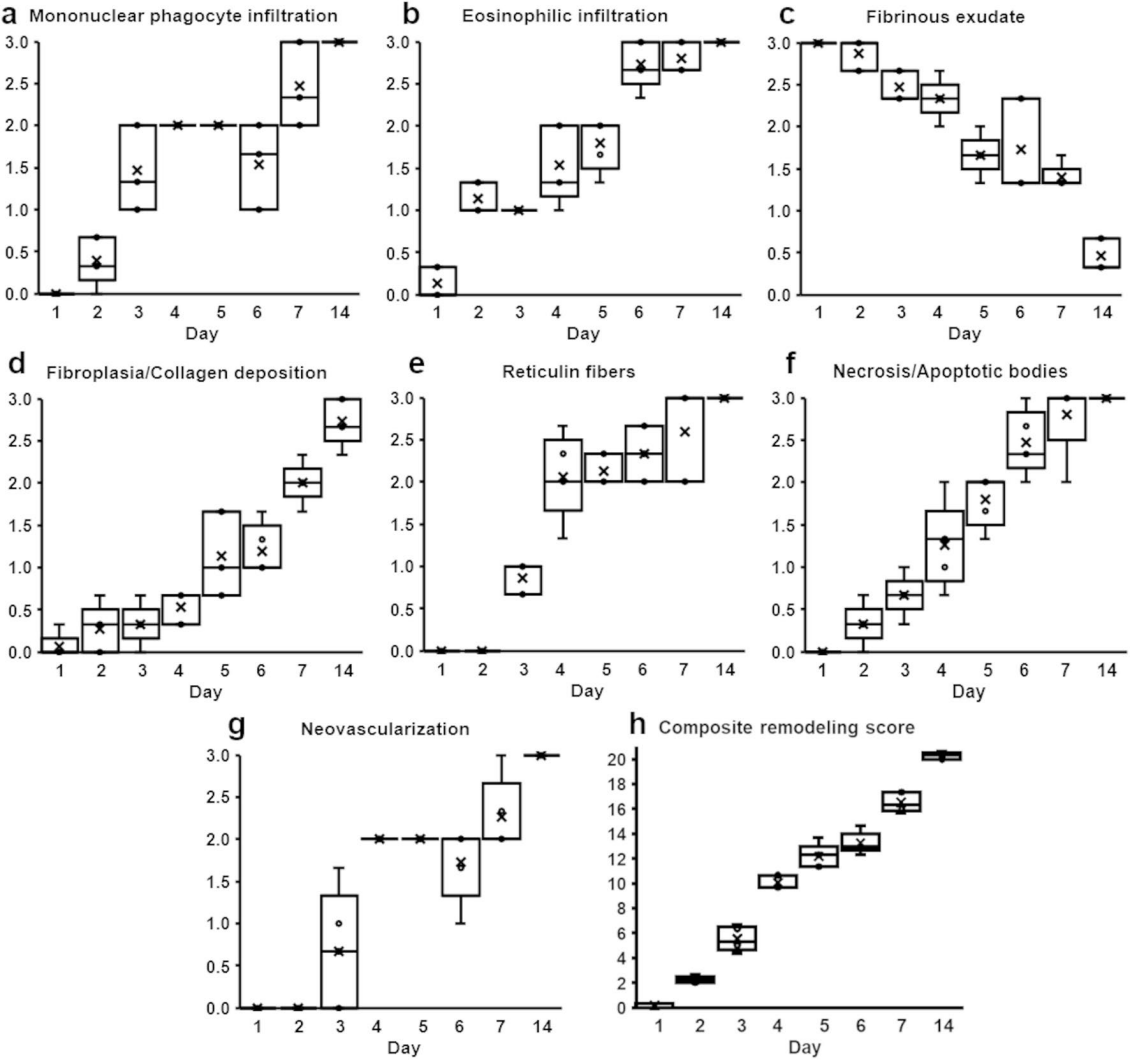
Semiquantitative histopathology scoring over time. Box-and-whisker plots show the distribution of per-animal scores (*n* = 5 animals/time point) for each histopathological feature across days 1, 2, 3, 4, 5, 6, 7, and 14. For each day, boxes represent the interquartile range (IQR) with the central line indicating the median; the “X” within the box indicates the mean; whiskers indicate the minimum and maximum values. Individual panels depict: **a** mononuclear phagocyte-rich infiltration, **b** eosinophil-rich infiltration, **c** fibrinous exudate/networks, **d** fibroplasia/collagen deposition and capsule maturation, **e** reticulin fiber organization, **f** necrosis/tissue degeneration and apoptotic bodies, **g** neovascularization, and **h** composite remodeling score. The composite score was computed per animal as the sum of feature scores after orienting all components to increase over time; because fibrin is expected to decline during remodeling, the fibrin score was inverted (3 − fibrin score) before summation

Mononuclear phagocyte-rich infiltration increased progressively from day 1 (0.00 [0.00–0.00]) and day 2 (0.33 [0.33–0.67]) to maximal values by day 14 (3.00 [3.00–3.00]) (Kruskal–Wallis *p* = 1.05 × 10^−5^) (Fig. 7a). Eosinophilic infiltration was detectable at day 1 (0.00 [0.00–0.33]) and increased thereafter, rising from day 2 (1.00 [1.00–1.33]) to day 7 (2.67 [2.67–3.00]) and peaking at day 14 (3.00 [3.00–3.00]) (Kruskal–Wallis *p* = 6.24 × 10^−6^) (Fig. 7b).

Fibrinous exudate/networks were maximal at the earliest stage (day 1, 3.00 [3.00–3.00]) and decreased across the time course, reaching low values by day 14 (0.67 [0.33–0.67]) (Kruskal–Wallis *p* = 1.16 × 10^−5^) (Fig. 7c). In contrast, fibroplasia/collagen deposition and capsule maturation increased from minimal levels at day 1 (0.00 [0.00–0.00]) to prominent late-stage remodeling at day 14 (2.67 [2.67–3.00]) (Kruskal–Wallis *p* = 1.14 × 10^−5^) (Fig. 7d). Reticulin fiber organization showed a delayed but marked increase, remaining absent at days 1–2 (0.00 [0.00–0.00] for both), increasing by day 3 (1.00 [0.67–1.00]), reaching maximal organization at day 14 (3.00 [3.00–3.00]) (Kruskal–Wallis *p* = 1.37 × 10^−5^) (Fig. 7e).

Necrosis/tissue degeneration and apoptotic bodies increased steadily from day 1 (0.00 [0.00–0.00]) and day 2 (0.33 [0.33–0.33]) to high scores by day 7 (3.00 [3.00–3.00]) and day 14 (3.00 [3.00–3.00]) (Kruskal–Wallis *p* = 5.86 × 10^−6^) (Fig. 7f). Neovascularization was absent at days 1–2 (0.00 [0.00–0.00] for both), increased at day 3 (0.67 [0.00–1.00]), and became prominent at later stages, reaching maximal values at day 14 (3.00 [3.00–3.00]) (Kruskal–Wallis *p* = 7.34 × 10^−6^) (Fig. 7g).

A composite remodeling score was computed per animal by summing oriented feature scores for each time point, with the fibrin component inverted (3 − fibrin score) to reflect its expected decline during remodeling; daily values were then summarized as median [IQR]. The composite score increased progressively from day 1 (0.33 [0.00–0.33]) to day 14 (20.33 [20.00–20.34]) (Kruskal–Wallis *p* = 2.77 × 10^−6^; Jonckheere–Terpstra *p* < 0.001), capturing the coordinated transition from early fibrin-rich exudation to fibrovascular capsule maturation (Fig. 7h).

### Association between eosinophil enrichment and remodeling features

Across animals, the eosinophil-rich infiltration score showed positive rank correlations with fibroplasia/collagen deposition, reticulin fiber organization, neovascularization, and necrosis/tissue degeneration and apoptotic bodies, and an inverse correlation with fibrinous exudate/networks (Supplementary Table S1). Because these variables also change systematically over time, we additionally evaluated partial rank correlations controlling for day. After controlling for day, most associations between eosinophil-rich infiltration and matrix remodeling features were markedly attenuated and no longer statistically significant (Supplementary Table S2). This indicates that the unadjusted correlations were largely driven by the coordinated temporal progression of eosinophil enrichment and lesion remodeling. Therefore, these analyses support temporal co-variation rather than time-independent or causal associations.

## Discussion

The temporal evolution from eosinophilic infiltration to organized fibrosis in this sterile granuloma model mirrors pathological transitions observed in human eosinophilic fibroses. This framework may support preclinical studies on the resolution of eosinophilic inflammation. To our knowledge, this is the first detailed, matrix-focused, compartmentalized histopathological time course describing the maturation of an eosinophil-enriched sterile implant granuloma in a sterile inflammatory model. The aim of this study was not to define cell-specific activation states or molecular mechanisms but to establish a temporal and compartmentalized histopathological framework of EWIp-induced sterile granuloma maturation.

The present study builds upon the findings of Vieira et al. (2021), who demonstrated that eosinophilic infiltration in the EWIp model is critically dependent on 5-LO activity and glucocorticoid signaling. Here, we have characterized the inflammatory response and granuloma formation induced by the intraperitoneal implantation of a heat-coagulated egg white pellet. Our findings document a dynamic progression of immune cell infiltration, extracellular matrix organization, and lesion compartmentalization, describing the spatial and temporal distribution of neutrophils, eosinophils, mononuclear phagocyte-rich areas, fibroplasia, and connective tissue maturation in response to a foreign protein deposit. The leukocyte infiltration followed a biphasic pattern, with an early neutrophilic response that was rapidly replaced by a sustained eosinophilic phase, ultimately contributing to granuloma maturation and tissue remodeling.

Although eosinophil recruitment in the EWIp model has been reported previously, the present work extends prior observations by providing a compartmentalized, matrix-focused histopathology atlas over a defined day 1–14 window and by integrating multiple extracellular matrix stains to map the ordered transition from fibrinous exudation to fibroplasia and collagen encapsulation. This time-resolved tissue architecture framework enables comparative evaluation of sterile granulomatous remodeling and provides standardized reference points for future mechanistic perturbations without altering the core model.

A key contribution of this study is the semiquantitative, compartment-focused scoring framework, which converted the descriptive time course into an interpretable summary of tissue composition transitions. Across days 1–14, the score captured the coordinated decline in fibrinous exudate and the progressive increase in fibroplasia/collagen deposition, reticulin organization, necrosis/apoptotic bodies, and neovascularization, while eosinophil enrichment persisted during the remodeling phase. This approach provides a standardized reference for comparing interventions or genetic backgrounds in future studies using the same model.

### Early acute inflammatory response: transient neutrophilia and the onset of eosinophilia

The initial phase of the immune response was characterized by a transient neutrophilic infiltrate surrounding the pellet, predominantly observed within the first 24 h. Neutrophils are well documented as the first line of defense against foreign materials, particularly in sterile inflammatory settings, where they respond to damage-associated molecular patterns (DAMPs) released from tissue injury and protein denaturation (Pittman and Kubes 2013). Despite their rapid influx, neutrophils were cleared from the inflammatory site by the second day, suggesting apoptosis and subsequent phagocytosis by macrophages (Greenlee-Wacker 2016). This early disappearance may indicate that neutrophils play a minimal role in sustaining the inflammatory response in this model, unlike their prolonged activity in bacterial infections or necrotic tissue environments.

As neutrophils declined, eosinophils rapidly accumulated, marking a shift in the inflammatory profile. By the second day, eosinophils were present in increasing numbers, and by the third day, they had become the predominant inflammatory cell type. This transition from neutrophilic to eosinophilic inflammation aligns with models of chronic immune responses to foreign proteins and persistent antigens (Xue et al. 2025). The extensive eosinophil degranulation observed, particularly in close proximity to the pellet, indicates local eosinophil activation within areas undergoing matrix alteration and pellet degradation. However, the present data do not establish whether eosinophils directly mediate these processes. The presence of apoptotic eosinophils, especially in proximity to fibrin deposits, indicates a regulated turnover, potentially driven by interactions with macrophages (Zustakova et al. 2020).

The cytological shift from early neutrophilia to late eosinophil enrichment in the peritoneal exudate, in parallel with the tissue-based scoring, reinforces a biphasic inflammatory profile coupled to progressive matrix remodeling.

### Granuloma formation: eosinophil persistence and macrophage-rich areas

By the fourth day, eosinophils dominated the inflammatory response, forming dense pericapsular infiltrates with persistent degranulation. This eosinophilic persistence is a key feature of chronic inflammatory responses to proteinaceous material and resembles patterns seen in parasitic infections and allergic reactions, where eosinophils contribute to both tissue destruction and remodeling (Macchia et al. 2023; Klimek et al. 2024). The emergence of fibroplasia and neovascularization at this stage occurred in parallel with persistent eosinophilic infiltration, indicating a spatial and temporal association between eosinophil enrichment and extracellular matrix remodeling. Direct involvement of eosinophils or specific cytokine pathways, however, remains to be tested experimentally (Allen 2023).

### Fibrin and collagen co-localization: transition to tissue repair

The presence of fibrin, particularly in the necrotic region, suggests the early stages of tissue repair and immune containment. Fibrin deposition plays a critical role in the acute phase of inflammation, acting as a scaffold for leukocyte infiltration and a barrier to pathogen spread (Luyendyk et al. 2019). The co-localization of fibrin and collagen, as evidenced by Masson’s trichrome, points to an important transitional phase between necrosis and healing. This dual presence of fibrin and collagen suggests a shift from an inflammatory response toward tissue remodeling, with fibrin initially acting as a provisional matrix that is gradually replaced by collagen as the granuloma matures.

### Macrophage-rich areas during granuloma organization

The presence of macrophages with morphology consistent with reduced activation/resolution in the outer zones of the granuloma could indicate a transition toward tissue repair and resolution (Watanabe et al. 2019). Because this interpretation is based solely on morphology, we have avoided assigning a defined activation phenotype or functional role to these cells. Their presence is therefore described as part of the histological transition toward lesion organization and connective tissue maturation. The presence of epithelioid macrophages and multinucleated giant cells, however, further supports this, as these cell types are characteristic of granulomatous inflammation, working together to encapsulate foreign material and contain the immune response (Chatterjee et al. 2021; Ahmadzadeh et al. 2022). This interpretation is based on morphology and would require immunophenotyping/functional assays for confirmation.

### Eosinophil persistence during late remodeling

By the seventh and 14th days, as a shift from an acute inflammatory phase to one of regulation and tissue remodeling occurred, eosinophils were observed in close association with macrophages and fibroblasts. At late time points, eosinophils remained detectable in areas undergoing fibroplasia and matrix organization. Because functional markers were not assessed, these findings should be interpreted as spatial and temporal associations. Defined eosinophil phenotypes should be explored in the future.

### Resolution phase and tissue remodeling

By the 14th day, the inflammatory response transitioned toward resolution, with the granuloma fully encapsulated by dense connective tissue. The previously distinct eosinophilic and necrotic layers merged into a single necroptotic zone, marked by abundant apoptotic bodies within a structurally degraded matrix. The persistence of eosinophils, though at reduced levels, in the outer layers of the granuloma suggests their involvement in the final stages of fibrosis and tissue repair. Notably, their association with macrophages and fibroblasts corroborates a shift from active inflammation to tissue remodeling (Levi-Schaffer et al. 1999; Dolgachev et al. 2008).

A significant observation was the presence of eosinophil-rich inflammatory changes in adjacent pancreatic tissue, suggesting that the inflammatory response extended beyond the primary granuloma site. Pancreatic involvement with eosinophil-rich inflammation has been reported in eosinophilic gastroenteropathies and hypereosinophilic syndromes, where excessive eosinophil infiltration leads to fibrosis and organ dysfunction (Sun et al. 2020). The marked structural damage and fibrosis in the pancreas observed in this study reinforce the notion that eosinophils, beyond their immunomodulatory roles, can contribute to pathology in sterile inflammatory environments.

### Comparative histopathological context of eosinophil-rich granulomatous lesions

The EWIp model shares several architectural features with eosinophil-rich fibroinflammatory lesions described in human pathology; however, we emphasize that these comparisons are intended as comparative context rather than direct disease validation.

In foreign body granulomas, eosinophils are frequently part of the early immune response to nondegradable materials. The presence of multinucleated giant cells and epithelioid macrophages surrounding the degenerating EWIp mirrors the reaction to biomaterials and parasitic remnants described in human tissues. Importantly, the capsule formation around the pellet, accompanied by collagen maturation and reticulin organization, resembles the structural evolution of human fibrotic encapsulation. This similarity positions the EWIp model as a useful surrogate for studying immune–matrix interactions in the absence of infection or antigenic variability.

Finally, eosinophilic fibrosing lesions, such as eosinophilic pancreatitis and sclerosing cholangitis, display similar histological hallmarks: dense eosinophilic infiltrates, degranulation, and progressive fibrosis (Noguchi et al. 1992). The advanced stages of the EWIp granuloma show some comparable histopathological features, including eosinophil-rich inflammation, apoptotic bodies, tissue degeneration, and collagen-rich encapsulation. The presence of co-localized fibrin and collagen in the late phase suggests a transitional matrix state analogous to the reparative fibrosis observed in these clinical entities.

In unadjusted rank analyses, higher eosinophil-rich infiltration scores co-varied with key matrix and repair features. These results strengthen the utility of the EWIp model as a standardized platform to interrogate immune–matrix relationships during sterile granuloma maturation, while also emphasizing that mechanistic causality will require targeted perturbation experiments.

This study has limitations. First, the study is primarily based on morphology and histochemical assessment. Therefore, although the temporal and spatial association between eosinophil enrichment and matrix remodeling is well documented, the underlying cell-biological mechanisms remain to be experimentally defined through targeted immunophenotyping, functional assays, and perturbation studies. The scoring framework is semiquantitative and morphology-based; although performed by independent observers, it does not substitute for immunophenotyping or functional assays to define leukocyte activation states. The EWIp model represents a highly standardized, artificial, sterile implant reaction. Although this controlled setting is useful for documenting reproducible temporal and spatial patterns of eosinophil-rich granulomatous inflammation and matrix remodeling, it does not capture the full complexity of chronic human fibroinflammatory diseases, which involve heterogeneous triggers, systemic immune regulation, tissue-specific factors, and disease-specific molecular pathways. Finally, mechanistic inferences regarding cytokine pathways or cell subpopulations remain testable hypotheses for future work.

Altogether, the EWIp model provides a controlled, antigen-dependent system that reproduces temporal and structural hallmarks of eosinophil-rich granulomatous inflammation. By documenting the spatial associations among eosinophils, macrophages, fibroplasia, neovascularization, and matrix organization, this model provides a useful histopathological platform for future mechanistic studies of chronic eosinophilic inflammation, foreign body reactions, and fibrosing granulomatous disorders.

## Supplementary Information

Below is the link to the electronic supplementary material.

Supplementary file1 (TIFF 15424 KB)

Supplementary file2 (TIFF 15608 KB)

Supplementary file3 (TIFF 15272 KB)

Supplementary file4 (DOCX 15 KB)

Supplementary file5 (DOCX 15 KB)

Supplementary file6 (DOCX 15 KB)

Supplementary file7 (DOCX 28 KB)

## Data Availability

Data is available on request from the authors.
