# Supplementary figures and images for: Eosinophil-associated matrix remodeling in a sterile granulomatous inflammation model: a temporal histopathological analysis
Source: Histochem Cell Biol. 2026 Jun 25;164(1):53. doi: 10.1007/s00418-026-02505-6 (PMC13303566; doi:10.1007/s00418-026-02505-6)

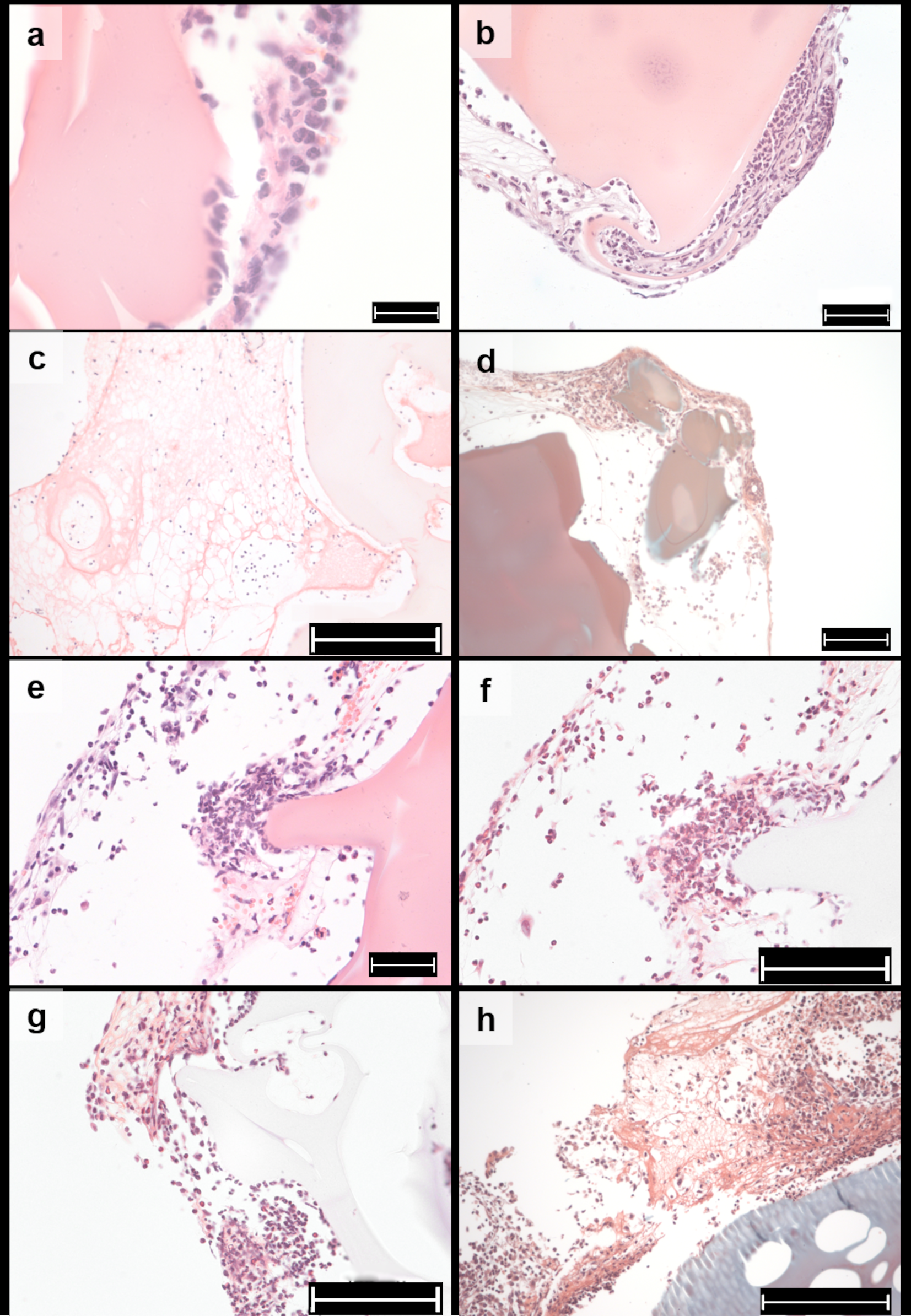

Supplement: Supplementary file 1 — Supplementary file1 (TIFF 15424 KB) [file 418_2026_2505_MOESM1_ESM.tiff]

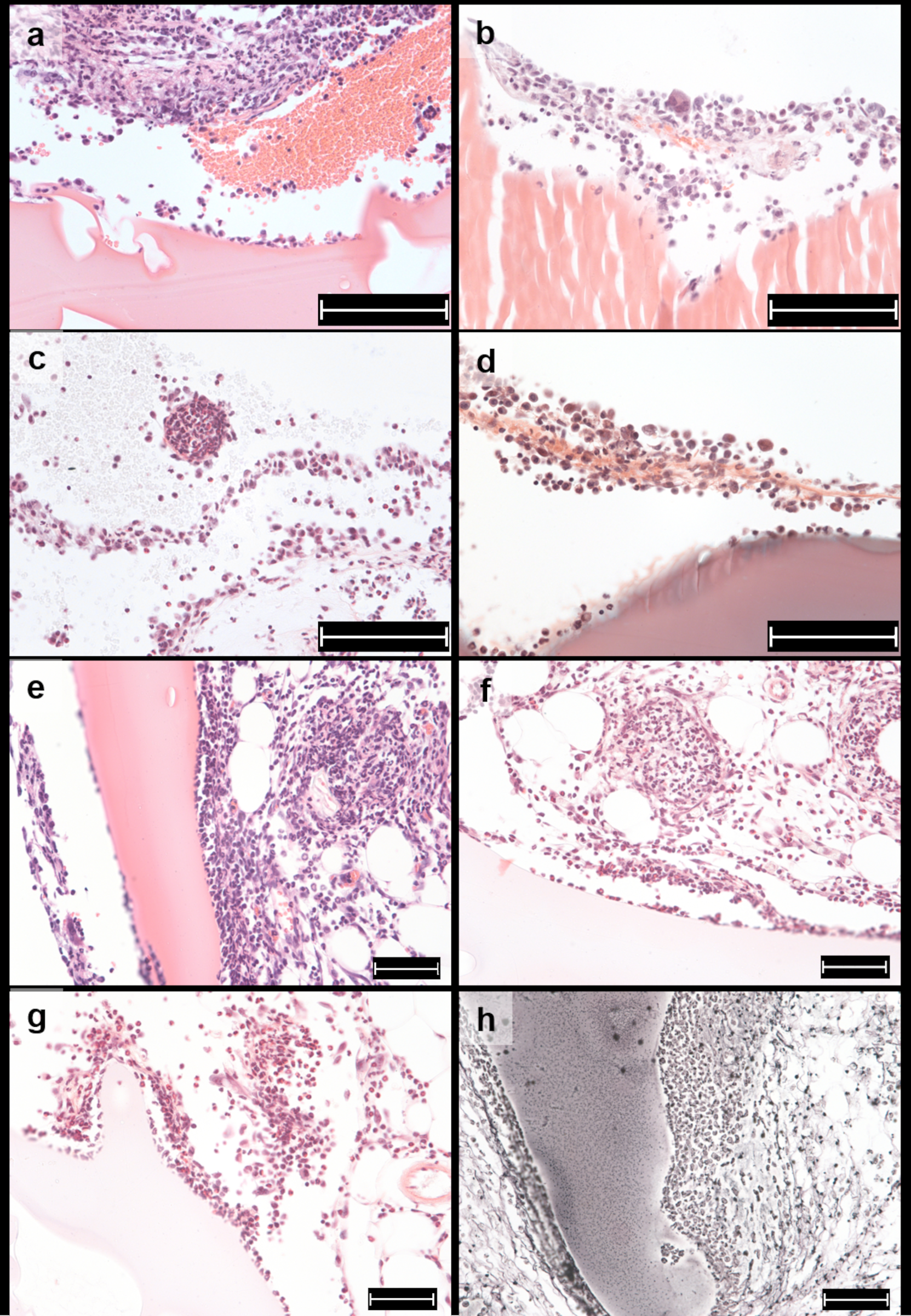

Supplement: Supplementary file 2 — Supplementary file2 (TIFF 15608 KB) [file 418_2026_2505_MOESM2_ESM.tiff]

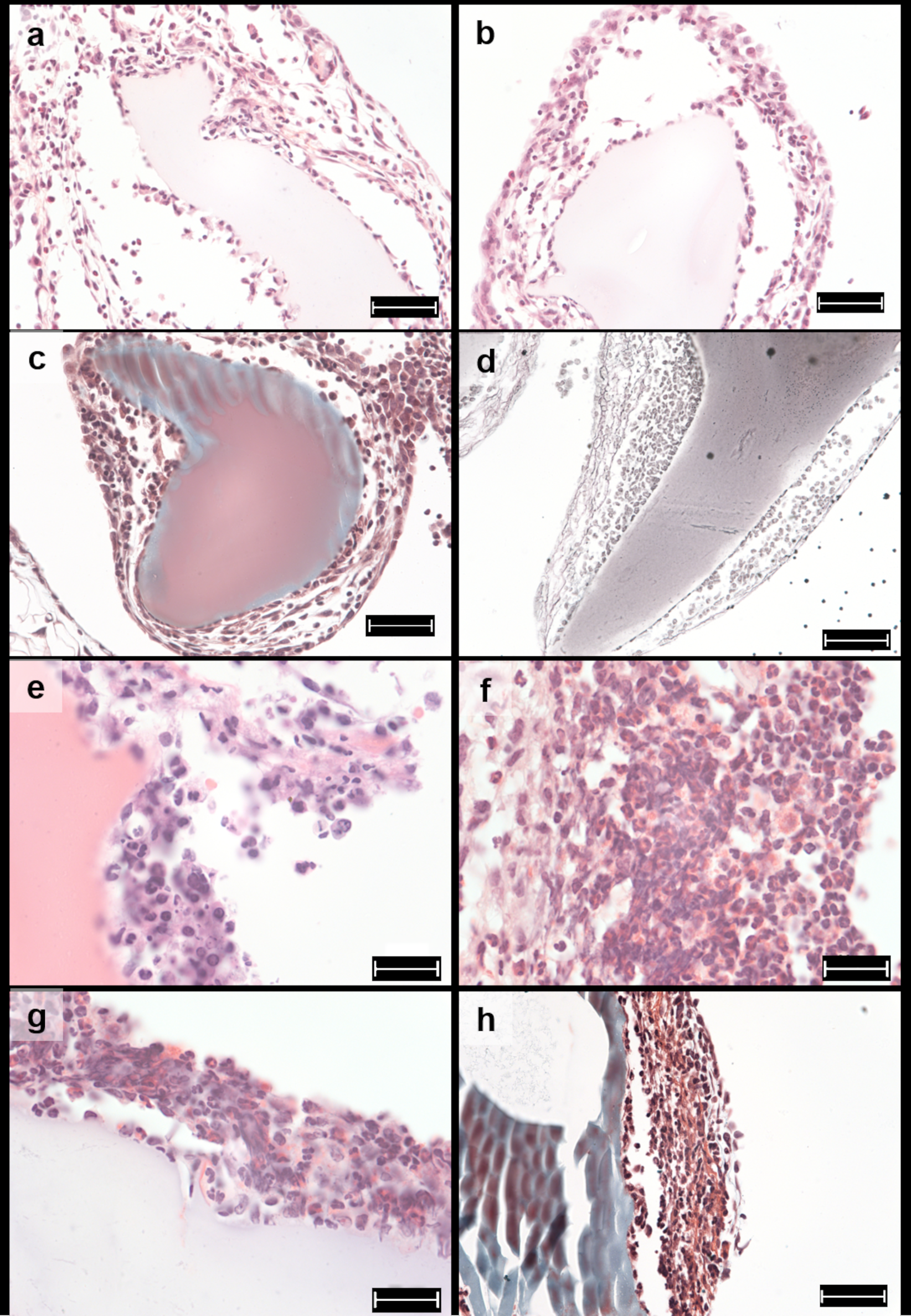

Supplement: Supplementary file 3 — Supplementary file3 (TIFF 15272 KB) [file 418_2026_2505_MOESM3_ESM.tiff]
