# Supplementary material for: Eosinophil-associated matrix remodeling in a sterile granulomatous inflammation model: a temporal histopathological analysis
Source: Histochem Cell Biol. 2026 Jun 25;164(1):53. doi: 10.1007/s00418-026-02505-6 (PMC13303566; doi:10.1007/s00418-026-02505-6)
Supplement: Supplementary file 4 — Supplementary file4 (DOCX 15 KB) [file 418_2026_2505_MOESM4_ESM.docx]

**Eosinophil-associated matrix remodeling in a sterile granulomatous inflammation model: a temporal histopathological analysis.**

**Histochemistry and Cell Biology**

**Bruno Marques Vieira; Milla Bezerra Paiva; Juliane Siqueira Francisco; Rebeca Sousa Brum; Lucas Everton Simões; Maria Ignez Capella Gaspar-Elsas; Pedro Xavier-Elsas**

**Supplementary Table S1. Unadjusted rank correlations between eosinophil enrichment and remodeling features.** Spearman rank correlation coefficients (ρ) and two-sided p values are shown for associations between the eosinophil-rich infiltration score and histopathological remodeling features (fibroplasia/collagen deposition and capsule maturation; reticulin fiber organization; neovascularization; necrosis/tissue degeneration and apoptotic bodies; fibrinous exudate/networks), as well as the composite remodeling score. Analyses used per-animal values pooled across all time points (n = 40 observations; 5 animals per day across 8 time points).

| **Comparator** | **Spearman ρ** | **p** |
| --- | --- | --- |
| Fibroplasia/collagen deposition & capsule maturation | 0.878229223 | 9.749E-14 |
| Reticulin fiber organization | 0.842785705 | 9.04728E-12 |
| Neovascularization | 0.825607286 | 5.53679E-11 |
| Necrosis, tissue degeneration & apoptotic bodies | 0.906962267 | 7.59124E-16 |
| Fibrinous exudate/networks | -0.856081728 | 1.90699E-12 |
| Composite remodeling score | 0.926529242 | 1.01735E-17 |
| Mononuclear phagocyte–rich infiltration | 0.733696296 | 7.20192E-08 |
