# Supplementary material for: Eosinophil-associated matrix remodeling in a sterile granulomatous inflammation model: a temporal histopathological analysis
Source: Histochem Cell Biol. 2026 Jun 25;164(1):53. doi: 10.1007/s00418-026-02505-6 (PMC13303566; doi:10.1007/s00418-026-02505-6)
Supplement: Supplementary file 5 — Supplementary file5 (DOCX 15 KB) [file 418_2026_2505_MOESM5_ESM.docx]

**Eosinophil-associated matrix remodeling in a sterile granulomatous inflammation model: a temporal histopathological analysis.**

**Histochemistry and Cell Biology**

**Bruno Marques Vieira; Milla Bezerra Paiva; Juliane Siqueira Francisco; Rebeca Sousa Brum; Lucas Everton Simões; Maria Ignez Capella Gaspar-Elsas; Pedro Xavier-Elsas**

**Supplementary Table S2. Partial rank correlations controlling for day (sensitivity analysis).** Partial rank correlation coefficients (ρ) and two-sided p values are shown for associations between the eosinophil-rich infiltration score and the same remodeling features as in Supplementary Table S1, after controlling for day as an ordered covariate (rank-based residual approach). This analysis was performed to reduce confounding by temporal progression in the time-course design (n = 40 observations).

| **Comparator** | **Partial Spearman ρ (Day)** | **p** |
| --- | --- | --- |
| Fibroplasia/collagen deposition & capsule maturation | 0.045703023 | 0.779453111 |
| Reticulin fiber organization | -0.268143039 | 0.094346619 |
| Neovascularization | -0.150180041 | 0.354984721 |
| Necrosis, tissue degeneration & apoptotic bodies | 0.062850941 | 0.70002945 |
| Fibrinous exudate/networks | 0.142884102 | 0.379104239 |
| Composite remodeling score | -0.054975219 | 0.736174644 |
| Mononuclear phagocyte–rich infiltration | -0.400680519 | 0.01040363 |
