# Supplementary material for: Eosinophil-associated matrix remodeling in a sterile granulomatous inflammation model: a temporal histopathological analysis
Source: Histochem Cell Biol. 2026 Jun 25;164(1):53. doi: 10.1007/s00418-026-02505-6 (PMC13303566; doi:10.1007/s00418-026-02505-6)
Supplement: Supplementary file 6 — Supplementary file6 (DOCX 15 KB) [file 418_2026_2505_MOESM6_ESM.docx]

**Eosinophil-associated matrix remodeling in a sterile granulomatous inflammation model: a temporal histopathological analysis.**

**Histochemistry and Cell Biology**

**Bruno Marques Vieira; Milla Bezerra Paiva; Juliane Siqueira Francisco; Rebeca Sousa Brum; Lucas Everton Simões; Maria Ignez Capella Gaspar-Elsas; Pedro Xavier-Elsas**

Supplementary Figure 1. Early inflammatory profile and matrix organization on Days 1 and 2. (a–d) Day 1 histological sections. (a–b) H&E: limited neutrophilic and eosinophilic infiltration adjacent to the pellet. (c) Sirius Red: scattered eosinophils within the early inflammatory infiltrate. (d) Masson’s Trichrome: absence of organized collagen deposition. (e–h) Day 2 histological sections. (e) H&E: increased eosinophilic and mononuclear cell infiltration. (f–g) Sirius Red: eosinophilic degranulation and apoptotic bodies surrounding the pellet. (h) Masson’s Trichrome: non-collagenous extracellular matrix with no structured fibroplasia. Scale bars: a=50 μm; b=50 μm; c=200 μm; d=100 μm; e=100 μm; f=200 μm; g=200 μm; h=200 μm.

Supplementary Figure 2. Eosinophilic dominance and early granuloma organization on Days 3 and 4. (a–d) Day 3 sections. (a) H&E: intense eosinophilic infiltration and early fibroplasia. (b) Sirius Red: eosinophil accumulation near the pellet and within the forming matrix. (c) Masson’s Trichrome: initial collagen deposition outlining the outer capsule. (d) Gomori’s Reticulin: thin reticular fibers at the granuloma periphery. (e–h) Day 4 sections. (e) H&E: dense eosinophilic infiltrate with apoptotic bodies and macrophages. (f–g) Sirius Red: strong eosinophilic accumulation and degranulation adjacent to the pellet, highlighting the predominance of eosinophils in the granulomatous tissue. (h) Gomori’s Reticulin: increased reticular fiber organization defining the nascent capsule. Scale bars: a=200 μm; b=100 μm; c=200 μm; d=100 μm; e=50 μm; f=50 μm; g=50 μm; h=50 μm.

Supplementary Figure 3. Granuloma maturation and matrix remodeling on Days 5 and 6. (a–d) Day 5 sections. (a–b) Sirius Red: apoptotic eosinophils with pyknotic nuclei near the capsule, showing active cellular turnover. (c) Masson’s Trichrome: collagen fibers in blue outlining the capsule and forming a fibrous layer at the periphery of the granuloma. (d) Gomori’s Reticulin: reticular fibers at the outer edge of the capsule, supporting the developing fibrous structure. (e–h) Day 6 sections. (e) H&E: necrotic region with polymorphonuclear cells, fibroblasts, and apoptotic bodies surrounding the pellet. (f) Sirius Red: eosinophilic infiltration penetrating the degenerating pellet. (g) Sirius Red: macrophage-like/mononuclear phagocyte-rich cells adjacent to apoptotic debris and mitotic figures, indicating ongoing inflammatory activity. (h) Masson’s Trichrome: fibrillar connective tissue degeneration in red among inflammatory cells at the periphery and intense collagen deposition in mononuclear regions. Scale bars: a=50 μm; b=50 μm; c=50 μm; d=50 μm; e=20 μm; f=20 μm; g=20 μm; h=50 μm.
