# Supplementary material for: Eosinophil-associated matrix remodeling in a sterile granulomatous inflammation model: a temporal histopathological analysis
Source: Histochem Cell Biol. 2026 Jun 25;164(1):53. doi: 10.1007/s00418-026-02505-6 (PMC13303566; doi:10.1007/s00418-026-02505-6)
Supplement: Supplementary file 7 — Supplementary file7 (DOCX 28 KB) [file 418_2026_2505_MOESM7_ESM.docx]

**Eosinophil-associated matrix remodeling in a sterile granulomatous inflammation model: a temporal histopathological analysis.**

**Histochemistry and Cell Biology**

**Bruno Marques Vieira; Milla Bezerra Paiva; Juliane Siqueira Francisco; Rebeca Sousa Brum; Lucas Everton Simões; Maria Ignez Capella Gaspar-Elsas; Pedro Xavier-Elsas**

**Supplementary Methods**

1. **Semi‑quantitative histopathology scoring (0–3)**

The following criteria were used for histopathological scoring.

| Feature | Stain | Score definition (0–3) |
| --- | --- | --- |
| Mononuclear phagocyte–rich infiltration | H&E | 0 none; 1 scattered mononuclear phagocytes; 2 multifocal moderate infiltrate; 3 dense, sheet-like or confluent infiltrate in the compartment |
| Eosinophilic infiltration | Sirius Red | 0 none; 1 few scattered eosinophils; 2 frequent eosinophils, with small focal clusters; 3 abundant/dense eosinophils, often forming aggregates or a band-like distribution at the interface |
| Fibrinous exudate/networks | Masson’s trichrome | 0 absent; 1 thin/discontinuous fibrin strands; 2 readily apparent networks occupying a substantial fraction of the compartment; 3 extensive, dense fibrin networks dominating the compartment |
| Fibroplasia/collagen deposition & capsule maturation | Picrosirius | 0 absent; 1 early fibroblast activation with sparse collagen; 2 moderate collagen bundles with developing capsule; 3 thick, continuous collagenous capsule/encapsulation with dense bundles |
| Reticulin fiber organization | Gomori’s reticulin | 0 absent; 1 sparse/disorganized fibers; 2 moderate fibers forming a partial framework; 3 dense, organized reticulin framework outlining capsule/lesion architecture |
| Neovascularization | H&E | 0 none; 1 occasional newly formed vessels; 2 frequent small vessels with focal leakage/erythrocytes; 3 prominent vascular proliferation throughout the compartment |
